# Di-μ-iodido-bis­{[(4-fluoro­benzoyl­methyl­ene)triphenyl-λ^5^-phospho­rane]iodido­mercury(II)}

**DOI:** 10.1107/S1600536808008611

**Published:** 2008-04-02

**Authors:** Mehmet Akkurt, Kazem Karami, Şerife Pınar Yalçın, Orhan Büyükgüngör

**Affiliations:** a Department of Physics, Faculty of Arts and Sciences, Erciyes University, 38039 Kayseri, Turkey; b Department of Chemistry, Isfahan University of Technology, Isfahan, 84156/83111, Iran; c Department of Physics, Faculty of Arts and Sciences, Ondokuz Mayıs University, 55139 Samsun, Turkey

## Abstract

In the title complex, [Hg_2_I_4_(C_26_H_20_FOP)_2_], the Hg^II^ centre is four-coordinate with one short Hg—I bond [2.6895 (7) Å], one Hg—C bond and two asymmetric bridging Hg—I bonds with distances of 2.7780 (8) and 3.2599 (8) Å. The title mol­ecule has a crystallographic inversion centre at the centroid of the four-membered ring formed by the two Hg atoms and two I atoms. The crystal packing is stabilized by C—H⋯O hydrogen bonds.

## Related literature

For related literature, see: Baenziger *et al.* (1978 ▶); Belluco *et al.* (1996 ▶); Bent (1961 ▶); Holy *et al.* (1976 ▶); Kalyanasundari *et al.* (1995 ▶, 1999 ▶); Karami (2007 ▶); Laavanya *et al.* (2001 ▶); Uson *et al.* (1985 ▶).
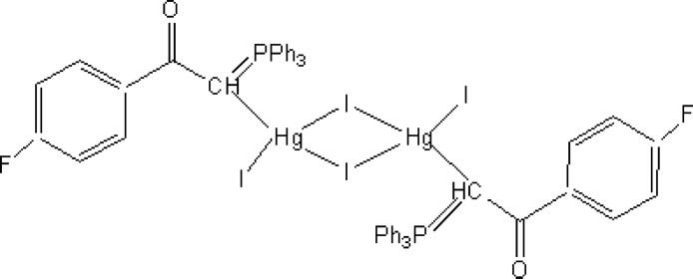


## Experimental

### 

#### Crystal data

[Hg_2_I_4_(C_26_H_20_FOP)_2_]
*M* *_r_* = 1705.56
Triclinic, 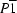

*a* = 10.0346 (16) Å
*b* = 11.8594 (19) Å
*c* = 13.235 (2) Å
α = 92.513 (13)°
β = 111.293 (12)°
γ = 113.117 (12)°
*V* = 1317.4 (4) Å^3^
*Z* = 1
Mo *K*α radiation
μ = 8.27 mm^−1^
*T* = 293 (2) K
0.26 × 0.17 × 0.08 mm

#### Data collection

Stoe IPDSII diffractometer
Absorption correction: integration (*X-RED32*; Stoe & Cie, 2002 ▶) *T*_min_ = 0.222, *T*_max_ = 0.558
16225 measured reflections
5553 independent reflections
4486 reflections with *I* > 2σ(*I*)
*R*_int_ = 0.156

#### Refinement

*R*[*F* ^2^ > 2σ(*F* ^2^)] = 0.045
*wR*(*F* ^2^) = 0.089
*S* = 1.05
5553 reflections
289 parameters
H-atom parameters constrained
Δρ_max_ = 1.00 e Å^−3^
Δρ_min_ = −0.67 e Å^−3^

### 

Data collection: *X-AREA* (Stoe & Cie, 2002 ▶); cell refinement: *X-AREA*; data reduction: *X-RED32* (Stoe & Cie, 2002 ▶); program(s) used to solve structure: *SIR97* (Altomare *et al.*, 1999 ▶); program(s) used to refine structure: *SHELXL97* (Sheldrick, 2008 ▶); molecular graphics: *ORTEP-3 for Windows* (Farrugia, 1997 ▶); software used to prepare material for publication: *WinGX* (Farrugia, 1999 ▶).

## Supplementary Material

Crystal structure: contains datablocks global, I. DOI: 10.1107/S1600536808008611/bt2690sup1.cif

Structure factors: contains datablocks I. DOI: 10.1107/S1600536808008611/bt2690Isup2.hkl

Additional supplementary materials:  crystallographic information; 3D view; checkCIF report

## Figures and Tables

**Table:** 

Hg1—I1	2.7780 (8)
Hg1—I2	2.6895 (7)
Hg1—C19	2.281 (5)
Hg1—I1^i^	3.2599 (8)

**Table:** 

I1—Hg1—I2	111.82 (2)
I1—Hg1—C19	116.49 (16)
I1—Hg1—I1^i^	94.17 (2)
I2—Hg1—C19	127.98 (15)
I1^i^—Hg1—I2	97.77 (2)
I1^i^—Hg1—C19	96.90 (16)
Hg1—I1—Hg1^i^	85.84 (2)

Symmetry code: (i) 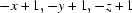.

**Table 2:** Hydrogen-bond geometry (Å, °)

*D*—H⋯*A*	*D*—H	H⋯*A*	*D*⋯*A*	*D*—H⋯*A*
C4—H4⋯O1^ii^	0.93	2.59	3.271 (11)	131
C12—H12⋯O1	0.93	2.32	3.124 (8)	144
C22—H22⋯O1	0.93	2.44	2.749 (10)	100

Symmetry code: (ii) 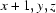.

**Fig. 1.**
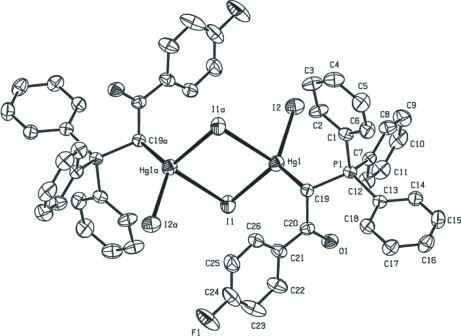
An ORTEP-3 view of the title compound with the atom numbering scheme. Displacement ellipsoids for non-H atoms are drawn at the 30% probability level.

**Fig. 2.**
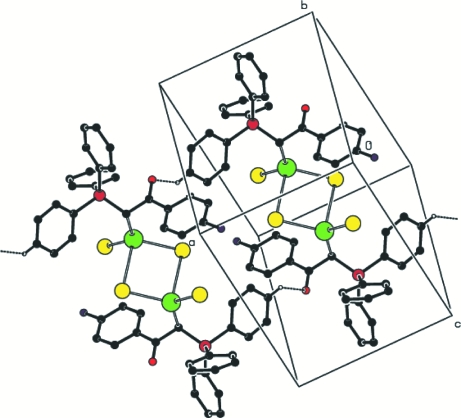
The packing and intermolecular hydrogen bonding interactions of the title compound.

## supplementary crystallographic information

### Comment

The dimeric structure adopted by complexes is in contrast to the O-coordinated trinuclear mercury (II) complex of the phosphorusylide Ph_3_PCHCOPh (Kalyanasundari *et al.*, 1999), but is similar to the structure of *trans*-di-liododiiodobis (triphenyl phosphoniumcyclopentadienylide) dimercury(II) reported by Baenziger *et al.* (Baenziger *et al.*, 1978) and the C-coordinated dinuclear mercury(II) halide complexes of Ph_3_CHCOPh(BPPY) (Kalyanasundari *et al.*, 1995). The C-coordination of FBPPY is in stark contrast to the O-coordination of the phosphorus ylide, Ph_3_PC(COMe)(COPh) (ABPPY), to a Hg^II^ centre (Laavanya *et al.*, 2001). The difference in coordination mode between ABPPY and FBPPY to Hg^II^ can be rationalized in terms of the electronic properties and steric requirements of the ylides. The nucleophilicity of the carbanion in ABPPY is less than for FBPPY; this is due to the additional delocalization of the ylide electron density in ABPPY which is facilitated by the second carbonyl group. This will reduce the ability of ABPPY to bind *via* the ylidic carbon. Belluco *et al.* have studied steric influences on the coordination modes of ylide molecules to Pt(II) systems (Belluco *et al.*, 1996). These authors concluded that the preferred coordination mode is *via* the ylidic carbon, but that steric hindrance around the metal centre or the ylidic carbon will necessitate O-coordination. Indeed, this trend is reflected here, both BPPY and FBPPY are slightly less sterically demanding than ABPPY, and both are C-coordinated to Hg^II^.

The title molecule has a crystallographic inversion symmety in the mid-point of the four-membered ring formed by the two Hg atoms and two I atoms (Fig.1). The crystal structure of the title complex reveals that the Hg^II^ centre forms four close contacts with *sp*^3^ hybridization and has a 4-coordinate environment with one short Hg—I bond 2.6895 (7), one Hg—C bond and two asymmetric bridging Hg—I bonds at distances of 2.7780 (8) and 3.2599 (8) Å in complex [[{HgI_2_(FBPPY)}_2_]. The significant shortening of the Hg—C bond length, 2.281 (5) Å compared to analogous distances in [(C_6_H_5_)_3_PCHCOC_6_H_5_HgI_2_]_2 _(Kalyanasundari *et al.*,1995) and in [(C_5_H_4_P(C_6_H_5_)_3_HgI_2_]_2 _ (Holy *et al.*, 1976) [2.312 (13) and 2.292 (8) Å, respectively] must be attributed to the use of mercury orbitals with high s character for bonding to the ylidic carbon. The use of non-equivalent hybrid orbitals with high s character to bond to low electronegative atoms was proposed by Bent in the concept of isovalent hybridization to account for the variation in bond lengths and bond angles around a central atom (Bent, 1961). The terminal Hg—I bond length, 2.7780 (8) Å is comparable to 2.615 Å observed in the case of Hg_2_l_4_(ABPPY)_2_, which has a tetrahedral coordination environment around mercury with a bridging structure (Laavanya *et al.*, 2001). The two bridged Hg—I bonds fall within the range 2.778 - 3.25994 Å reported for other structures (Laavanya *et al.*, 2001) containing chloro bridged mercury. The angles around mercury vary from 94.17 (2) to111.82 (2) for I—Hg—I, a very distorted tetrahedral environment. This distortion must be due to the higher s character of the *sp*^3^ hybrid mercury orbitals involved in the above bonds and the formation of a strong Iodo bridge between the Hg atoms which requires the internal I—Hg—I angle to be considerably smaller. The stabilized resonance structure for the title ylide is destroyed by the complexes formation. On the other hand, the bond length of P(1)—C(19) in the similar ylide is 1.706 Å (Uson *et al.*, 1985) which shows that the above bond is considerably elongated to 1.787 (6) Å in complex [{HgI_2_(FBPPY)}_2_]. The adaptation of dimeric structur in Hg^II^ ylide complex may be explained by both the preference of Hg^II^ to four coordination and the stability of the 18 electron configuration around Hg^II^.

### Experimental

To a chloroform solution (15 ml) of triphenylphosphine (1 mmol) was added 2-bromo-4-fluoroacetophenone (1 mmol) and the resulting mixture was stirred for 12 h. The solution was filtered off, and the precipitate washed with diethyl-ether and air-dried. Further treatment with aqueous NaOH solution (0.5*M*) led to elimination of HBr, giving the free ligand precursors FBPPY. To a solution of FBPPY (0.100 g, 0.25 mmol) in acetone (5 ml) was added mercury (II) iodide (0.114 g, 0.25 mmol). The mixture was stirred for 12 h. On concentration by removing the solvent by vacuum, a pale yellow precipitate was obtained. The products were washed with benzene and dried *in vacuo*. Yield: 81%, *M*.p. 214 °C. Analysis calculated for C_52_H_40_F_2_Hg_2_I_4_O_2_P_2_:*C* 36.6, H 2.4%. Found: C 36.45, H 2.3%, ^1^H NMR: 4.62(d, 1H, CH, 2*J*PH= 5.5 Hz), 7.1–8 (m, 19H, Ph) p.p.m. and ^31^P NMR: 20.34 p.p.m. (Karami, 2007).

### Refinement

H atoms were placed in calculated positions and refined using a riding model with C—H = 0.93 Å and *U*_iso_(H) = 1.2*U*_eq_(C).

### Crystal data

**Table:**

[Hg_2_I_4_(C_26_H_20_FOP)_2_]	*Z* = 1
*M**_r_* = 1705.56	*F*_000_ = 788
Triclinic, *P*1	*D*_x_ = 2.150 Mg m^−^^3^
Hall symbol: -P 1	Mo *K*α radiation λ = 0.71073 Å
*a* = 10.0346 (16) Å	Cell parameters from 47681 reflections
*b* = 11.8594 (19) Å	θ = 1.7–28.3º
*c* = 13.235 (2) Å	µ = 8.27 mm^−^^1^
α = 92.513 (13)º	*T* = 293 (2) K
β = 111.293 (12)º	Plate, colourless
γ = 113.117 (12)º	0.26 × 0.17 × 0.08 mm
*V* = 1317.4 (4) Å^3^	

### Data collection

**Table:**

Stoe IPDSII diffractometer	5553 independent reflections
Monochromator: plane graphite	4486 reflections with *I* > 2σ(*I*)
Detector resolution: 6.67 pixels mm^-1^	*R*_int_ = 0.156
*T* = 293(2) K	θ_max_ = 27.0º
ω scans	θ_min_ = 1.7º
Absorption correction: integration(X-RED32; Stoe & Cie, 2002)	*h* = −12→12
*T*_min_ = 0.222, *T*_max_ = 0.558	*k* = −15→15
16225 measured reflections	*l* = −16→16

### Refinement

**Table:**

Refinement on *F*^2^	Secondary atom site location: difference Fourier map
Least-squares matrix: full	Hydrogen site location: inferred from neighbouring sites
*R*[*F*^2^ > 2σ(*F*^2^)] = 0.045	H-atom parameters constrained
*wR*(*F*^2^) = 0.089	*w* = 1/[σ^2^(*F*_o_^2^) + (0.0388*P*)^2^ + 0.7953*P*] where *P* = (*F*_o_^2^ + 2*F*_c_^2^)/3
*S* = 1.05	(Δ/σ)_max_ = 0.001
5553 reflections	Δρ_max_ = 1.00 e Å^−^^3^
289 parameters	Δρ_min_ = −0.67 e Å^−^^3^
Primary atom site location: structure-invariant direct methods	Extinction correction: none

### Special details

**Table:**

Geometry. Bond distances, angles *etc*. have been calculated using the rounded fractional coordinates. All su's are estimated from the variances of the (full) variance-covariance matrix. The cell e.s.d.'s are taken into account in the estimation of distances, angles and torsion angles
Refinement. Refinement on *F*^2^ for ALL reflections except those flagged by the user for potential systematic errors. Weighted *R*-factors *wR* and all goodnesses of fit *S* are based on *F*^2^, conventional *R*-factors *R* are based on *F*, with *F* set to zero for negative *F*^2^. The observed criterion of *F*^2^ > σ(*F*^2^) is used only for calculating -*R*-factor-obs *etc*. and is not relevant to the choice of reflections for refinement. *R*-factors based on *F*^2^ are statistically about twice as large as those based on *F*, and *R*-factors based on ALL data will be even larger.

### Fractional atomic coordinates and isotropic or equivalent isotropic displacement parameters (Å^2^)

**Table:**

	*x*	*y*	*z*	*U*_iso_*/*U*_eq_	
Hg1	0.53557 (3)	0.65438 (3)	0.42856 (2)	0.0581 (1)	
I1	0.29177 (6)	0.52860 (4)	0.49338 (4)	0.0650 (2)	
I2	0.76379 (6)	0.86528 (5)	0.57953 (4)	0.0727 (2)	
P1	0.58806 (17)	0.74049 (12)	0.20232 (11)	0.0389 (4)	
F1	−0.1102 (8)	0.0461 (5)	0.0631 (6)	0.133 (3)	
O1	0.2430 (5)	0.6313 (4)	0.1541 (4)	0.0613 (16)	
C1	0.7783 (7)	0.7396 (5)	0.2330 (5)	0.0445 (17)	
C2	0.8580 (8)	0.7185 (7)	0.3349 (5)	0.062 (2)	
C3	0.9949 (9)	0.7038 (8)	0.3566 (6)	0.069 (3)	
C4	1.0532 (9)	0.7126 (8)	0.2760 (7)	0.074 (3)	
C5	0.9746 (10)	0.7344 (9)	0.1756 (7)	0.080 (3)	
C6	0.8373 (9)	0.7470 (7)	0.1524 (7)	0.065 (3)	
C7	0.6204 (7)	0.8894 (5)	0.2717 (5)	0.0442 (17)	
C8	0.7676 (8)	0.9929 (6)	0.3076 (6)	0.061 (2)	
C9	0.7889 (11)	1.1059 (6)	0.3622 (7)	0.076 (3)	
C10	0.6674 (12)	1.1146 (7)	0.3787 (6)	0.078 (3)	
C11	0.5234 (11)	1.0153 (7)	0.3426 (7)	0.072 (3)	
C12	0.4974 (9)	0.9025 (6)	0.2876 (6)	0.061 (2)	
C13	0.4969 (7)	0.7270 (5)	0.0543 (5)	0.0423 (17)	
C14	0.5281 (8)	0.8339 (6)	0.0109 (5)	0.0548 (19)	
C15	0.4683 (10)	0.8238 (7)	−0.1016 (6)	0.068 (3)	
C16	0.3754 (10)	0.7082 (7)	−0.1731 (6)	0.069 (2)	
C17	0.3410 (9)	0.6005 (6)	−0.1319 (5)	0.061 (2)	
C18	0.4023 (8)	0.6097 (5)	−0.0176 (5)	0.0527 (19)	
C19	0.4769 (6)	0.6070 (5)	0.2440 (4)	0.0401 (17)	
C20	0.3012 (7)	0.5596 (6)	0.1835 (5)	0.0463 (17)	
C21	0.1984 (7)	0.4217 (6)	0.1586 (5)	0.0539 (19)	
C22	0.0358 (9)	0.3826 (8)	0.1146 (8)	0.078 (3)	
C23	−0.0692 (11)	0.2564 (10)	0.0825 (10)	0.107 (4)	
C24	−0.0083 (12)	0.1715 (8)	0.0951 (8)	0.088 (3)	
C25	0.1511 (11)	0.2031 (7)	0.1366 (7)	0.081 (3)	
C26	0.2542 (8)	0.3310 (6)	0.1691 (6)	0.063 (2)	
H2	0.81880	0.71420	0.38910	0.0740*	
H3	1.04710	0.68820	0.42450	0.0830*	
H4	1.14600	0.70380	0.28970	0.0890*	
H5	1.01540	0.74060	0.12210	0.0960*	
H6	0.78430	0.76050	0.08360	0.0780*	
H8	0.85050	0.98690	0.29550	0.0730*	
H9	0.88720	1.17580	0.38750	0.0900*	
H10	0.68370	1.19050	0.41570	0.0940*	
H11	0.44150	1.02310	0.35490	0.0860*	
H12	0.39720	0.83480	0.26100	0.0730*	
H14	0.59000	0.91280	0.05830	0.0650*	
H15	0.49070	0.89610	−0.13000	0.0820*	
H16	0.33570	0.70260	−0.24940	0.0820*	
H17	0.27730	0.52210	−0.18020	0.0730*	
H18	0.38010	0.53740	0.01070	0.0630*	
H19	0.50550	0.53940	0.23070	0.0480*	
H22	−0.00370	0.44230	0.10640	0.0940*	
H23	−0.17860	0.23040	0.05300	0.1290*	
H25	0.18880	0.14240	0.14280	0.0970*	
H26	0.36340	0.35610	0.19860	0.0750*	

### Atomic displacement parameters (Å^2^)

**Table:**

	*U*^11^	*U*^22^	*U*^33^	*U*^12^	*U*^13^	*U*^23^
Hg1	0.0633 (2)	0.0652 (2)	0.0484 (1)	0.0285 (1)	0.0256 (1)	0.0120 (1)
I1	0.0709 (3)	0.0725 (3)	0.0830 (3)	0.0449 (2)	0.0483 (3)	0.0334 (2)
I2	0.0717 (3)	0.0778 (3)	0.0580 (3)	0.0308 (2)	0.0200 (2)	−0.0026 (2)
P1	0.0372 (7)	0.0383 (7)	0.0415 (7)	0.0166 (6)	0.0172 (6)	0.0033 (5)
F1	0.110 (4)	0.067 (3)	0.161 (6)	−0.022 (3)	0.062 (4)	−0.009 (3)
O1	0.049 (2)	0.065 (3)	0.074 (3)	0.033 (2)	0.021 (2)	0.015 (2)
C1	0.043 (3)	0.039 (3)	0.045 (3)	0.014 (2)	0.017 (2)	−0.002 (2)
C2	0.050 (4)	0.094 (5)	0.036 (3)	0.036 (3)	0.010 (3)	−0.003 (3)
C3	0.054 (4)	0.094 (5)	0.049 (4)	0.037 (4)	0.009 (3)	−0.005 (3)
C4	0.050 (4)	0.094 (5)	0.079 (5)	0.036 (4)	0.025 (4)	0.003 (4)
C5	0.077 (5)	0.120 (7)	0.084 (5)	0.056 (5)	0.059 (5)	0.041 (5)
C6	0.066 (4)	0.083 (5)	0.070 (4)	0.040 (4)	0.043 (4)	0.030 (3)
C7	0.048 (3)	0.046 (3)	0.044 (3)	0.027 (3)	0.018 (2)	0.008 (2)
C8	0.057 (4)	0.056 (4)	0.055 (4)	0.024 (3)	0.010 (3)	−0.007 (3)
C9	0.078 (5)	0.045 (4)	0.081 (5)	0.019 (3)	0.020 (4)	−0.006 (3)
C10	0.110 (7)	0.061 (4)	0.067 (4)	0.059 (5)	0.019 (4)	−0.001 (3)
C11	0.089 (6)	0.071 (5)	0.074 (5)	0.051 (4)	0.036 (4)	0.007 (4)
C12	0.068 (4)	0.058 (4)	0.070 (4)	0.036 (3)	0.033 (4)	0.011 (3)
C13	0.041 (3)	0.043 (3)	0.046 (3)	0.021 (2)	0.019 (2)	0.005 (2)
C14	0.057 (4)	0.048 (3)	0.049 (3)	0.018 (3)	0.017 (3)	0.007 (2)
C15	0.088 (5)	0.059 (4)	0.059 (4)	0.031 (4)	0.033 (4)	0.022 (3)
C16	0.090 (5)	0.074 (4)	0.042 (3)	0.044 (4)	0.019 (3)	0.009 (3)
C17	0.073 (4)	0.058 (4)	0.045 (3)	0.031 (3)	0.015 (3)	−0.002 (3)
C18	0.060 (4)	0.038 (3)	0.055 (3)	0.020 (3)	0.021 (3)	0.002 (2)
C19	0.040 (3)	0.043 (3)	0.041 (3)	0.019 (2)	0.020 (2)	0.008 (2)
C20	0.041 (3)	0.056 (3)	0.042 (3)	0.022 (3)	0.017 (2)	0.007 (2)
C21	0.045 (3)	0.059 (4)	0.048 (3)	0.011 (3)	0.023 (3)	0.003 (3)
C22	0.048 (4)	0.074 (5)	0.105 (6)	0.017 (4)	0.036 (4)	0.002 (4)
C23	0.052 (5)	0.098 (7)	0.136 (9)	−0.001 (5)	0.043 (5)	−0.018 (6)
C24	0.088 (6)	0.061 (5)	0.082 (5)	−0.008 (5)	0.046 (5)	−0.001 (4)
C25	0.087 (6)	0.052 (4)	0.077 (5)	0.012 (4)	0.025 (4)	0.012 (3)
C26	0.052 (4)	0.054 (4)	0.059 (4)	0.010 (3)	0.014 (3)	0.007 (3)

### Geometric parameters (Å, °)

**Table:**

Hg1—I1	2.7780 (8)	C19—C20	1.491 (10)
Hg1—I2	2.6895 (7)	C20—C21	1.493 (9)
Hg1—C19	2.281 (5)	C21—C22	1.382 (13)
Hg1—I1^i^	3.2599 (8)	C21—C26	1.385 (11)
P1—C1	1.806 (8)	C22—C23	1.383 (15)
P1—C7	1.805 (6)	C23—C24	1.356 (17)
P1—C13	1.805 (6)	C24—C25	1.369 (17)
P1—C19	1.787 (6)	C25—C26	1.394 (11)
F1—C24	1.369 (11)	C2—H2	0.9300
O1—C20	1.212 (9)	C3—H3	0.9300
C1—C2	1.387 (9)	C4—H4	0.9300
C1—C6	1.387 (12)	C5—H5	0.9300
C2—C3	1.381 (13)	C6—H6	0.9300
C3—C4	1.381 (13)	C8—H8	0.9300
C4—C5	1.373 (12)	C9—H9	0.9300
C5—C6	1.369 (15)	C10—H10	0.9300
C7—C8	1.389 (10)	C11—H11	0.9300
C7—C12	1.389 (13)	C12—H12	0.9300
C8—C9	1.394 (11)	C14—H14	0.9300
C9—C10	1.355 (17)	C15—H15	0.9300
C10—C11	1.348 (14)	C16—H16	0.9300
C11—C12	1.375 (11)	C17—H17	0.9300
C13—C14	1.385 (9)	C18—H18	0.9300
C13—C18	1.390 (8)	C19—H19	0.9800
C14—C15	1.368 (9)	C22—H22	0.9300
C15—C16	1.373 (11)	C23—H23	0.9300
C16—C17	1.378 (10)	C25—H25	0.9300
C17—C18	1.391 (9)	C26—H26	0.9300
			
Hg1···C2	3.694 (9)	C15···H23^x^	3.0300
Hg1···C12	3.624 (7)	C19···H2	2.9400
Hg1···C26	4.216 (7)	C19···H18	2.8500
Hg1···H2	2.8900	C19···H26	2.6800
Hg1···H12	3.4500	C20···H12	3.0300
Hg1···H26	3.9000	C20···H18	2.7100
I2···C25^i^	3.739 (8)	C23···H5^ix^	2.9500
I2···C7	3.863 (6)	C24···H5^ix^	3.0900
I1···H2^i^	3.3300	C26···H19	2.5600
I1···H10^ii^	3.3800	H2···Hg1	2.8900
I2···H2	3.3600	H2···I2	3.3600
I2···H8^iii^	3.2500	H2···C19	2.9400
I2···H11^ii^	3.1800	H2···I1^i^	3.3300
F1···C14^iv^	3.292 (11)	H3···C9^iii^	3.0800
F1···H14^iv^	2.7700	H3···C10^iii^	3.0400
O1···C4^v^	3.271 (11)	H3···H10^iii^	2.5200
O1···C12	3.124 (8)	H4···O1^vi^	2.5900
O1···C13	3.135 (9)	H4···H12^vi^	2.5700
O1···C18	3.270 (9)	H5···C23^ix^	2.9500
O1···H4^v^	2.5900	H5···C24^ix^	3.0900
O1···H12	2.3200	H6···C13	2.6300
O1···H22	2.4400	H6···C14	2.8900
C2···Hg1	3.694 (9)	H8···C1	2.7400
C4···O1^vi^	3.271 (11)	H8···I2^iii^	3.2500
C6···C14	3.558 (12)	H10···I1^ii^	3.3800
C7···I2	3.863 (6)	H10···H3^iii^	2.5200
C10···C16^vii^	3.517 (11)	H10···H16^vii^	2.5700
C12···O1	3.124 (8)	H11···I2^ii^	3.1800
C12···Hg1	3.624 (7)	H12···Hg1	3.4500
C13···O1	3.135 (9)	H12···O1	2.3200
C14···C6	3.558 (12)	H12···C20	3.0300
C14···F1^viii^	3.292 (11)	H12···H4^v^	2.5700
C16···C10^vii^	3.517 (11)	H14···F1^viii^	2.7700
C18···C20	3.177 (10)	H14···C7	2.7700
C18···O1	3.270 (9)	H14···C8	3.0200
C20···C18	3.177 (10)	H15···C10^vii^	3.0600
C25···I2^i^	3.739 (8)	H15···C11^vii^	3.0200
C26···Hg1	4.216 (7)	H16···C10^vii^	2.8200
C1···H17^ix^	2.9300	H16···H10^vii^	2.5700
C1···H8	2.7400	H17···C1^ix^	2.9300
C2···H19	3.0400	H17···C2^ix^	2.9300
C2···H17^ix^	2.9300	H17···C3^ix^	3.0300
C3···H17^ix^	3.0300	H17···C6^ix^	3.0300
C6···H17^ix^	3.0300	H18···C19	2.8500
C7···H14	2.7700	H18···C20	2.7100
C8···H14	3.0200	H19···C2	3.0400
C9···H3^iii^	3.0800	H19···C26	2.5600
C10···H15^vii^	3.0600	H19···H26	2.0000
C10···H16^vii^	2.8200	H22···O1	2.4400
C10···H3^iii^	3.0400	H23···C14^x^	3.0500
C11···H15^vii^	3.0200	H23···C15^x^	3.0300
C13···H6	2.6300	H26···Hg1	3.9000
C14···H23^x^	3.0500	H26···C19	2.6800
C14···H6	2.8900	H26···H19	2.0000
			
I1—Hg1—I2	111.82 (2)	C22—C23—C24	118.2 (11)
I1—Hg1—C19	116.49 (16)	F1—C24—C23	119.1 (11)
I1—Hg1—I1^i^	94.17 (2)	F1—C24—C25	116.9 (9)
I2—Hg1—C19	127.98 (15)	C23—C24—C25	124.0 (9)
I1^i^—Hg1—I2	97.77 (2)	C24—C25—C26	116.6 (9)
I1^i^—Hg1—C19	96.90 (16)	C21—C26—C25	121.8 (8)
Hg1—I1—Hg1^i^	85.84 (2)	C1—C2—H2	120.00
C1—P1—C7	109.1 (3)	C3—C2—H2	120.00
C1—P1—C13	106.6 (3)	C2—C3—H3	121.00
C1—P1—C19	106.1 (3)	C4—C3—H3	120.00
C7—P1—C13	108.4 (3)	C3—C4—H4	120.00
C7—P1—C19	114.7 (3)	C5—C4—H4	120.00
C13—P1—C19	111.6 (3)	C4—C5—H5	119.00
P1—C1—C2	119.1 (6)	C6—C5—H5	119.00
P1—C1—C6	120.8 (6)	C1—C6—H6	120.00
C2—C1—C6	119.7 (8)	C5—C6—H6	121.00
C1—C2—C3	120.7 (7)	C7—C8—H8	121.00
C2—C3—C4	119.0 (7)	C9—C8—H8	121.00
C3—C4—C5	120.1 (10)	C8—C9—H9	120.00
C4—C5—C6	121.4 (9)	C10—C9—H9	120.00
C1—C6—C5	119.1 (8)	C9—C10—H10	119.00
P1—C7—C8	120.4 (6)	C11—C10—H10	119.00
P1—C7—C12	120.4 (5)	C10—C11—H11	120.00
C8—C7—C12	119.2 (6)	C12—C11—H11	120.00
C7—C8—C9	118.8 (8)	C7—C12—H12	120.00
C8—C9—C10	120.4 (8)	C11—C12—H12	120.00
C9—C10—C11	121.3 (8)	C13—C14—H14	120.00
C10—C11—C12	120.1 (11)	C15—C14—H14	120.00
C7—C12—C11	120.2 (8)	C14—C15—H15	120.00
P1—C13—C14	120.0 (5)	C16—C15—H15	120.00
P1—C13—C18	120.6 (4)	C15—C16—H16	120.00
C14—C13—C18	119.3 (6)	C17—C16—H16	120.00
C13—C14—C15	120.2 (6)	C16—C17—H17	120.00
C14—C15—C16	120.8 (7)	C18—C17—H17	120.00
C15—C16—C17	120.1 (7)	C13—C18—H18	120.00
C16—C17—C18	119.6 (6)	C17—C18—H18	120.00
C13—C18—C17	120.0 (5)	Hg1—C19—H19	109.00
Hg1—C19—P1	110.7 (3)	P1—C19—H19	109.00
Hg1—C19—C20	106.7 (4)	C20—C19—H19	109.00
P1—C19—C20	113.5 (4)	C21—C22—H22	119.00
O1—C20—C19	120.8 (6)	C23—C22—H22	119.00
O1—C20—C21	120.6 (7)	C22—C23—H23	121.00
C19—C20—C21	118.6 (6)	C24—C23—H23	121.00
C20—C21—C22	117.0 (7)	C24—C25—H25	122.00
C20—C21—C26	124.6 (7)	C26—C25—H25	122.00
C22—C21—C26	118.3 (7)	C21—C26—H26	119.00
C21—C22—C23	121.2 (9)	C25—C26—H26	119.00
			
I2—Hg1—I1—Hg1^i^	−100.09 (2)	P1—C1—C2—C3	173.0 (6)
C19—Hg1—I1—Hg1^i^	99.83 (17)	C1—C2—C3—C4	1.1 (12)
I1^i^—Hg1—I1—Hg1^i^	0.00 (4)	C2—C3—C4—C5	−0.7 (13)
I1^i^—Hg1^i^—I1—Hg1	0.00 (5)	C3—C4—C5—C6	−0.5 (14)
I2^i^—Hg1^i^—I1—Hg1	−112.71 (2)	C4—C5—C6—C1	1.1 (13)
C19^i^—Hg1^i^—I1—Hg1	117.34 (16)	C8—C7—C12—C11	−2.8 (10)
I2—Hg1—C19—C20	−131.8 (3)	P1—C7—C8—C9	−179.1 (6)
I1^i^—Hg1—C19—C20	122.7 (4)	P1—C7—C12—C11	178.6 (6)
I2—Hg1—C19—P1	−7.9 (4)	C12—C7—C8—C9	2.3 (10)
I1^i^—Hg1—C19—P1	−113.4 (3)	C7—C8—C9—C10	−0.7 (11)
I1—Hg1—C19—P1	148.4 (2)	C8—C9—C10—C11	−0.4 (12)
I1—Hg1—C19—C20	24.6 (4)	C9—C10—C11—C12	−0.2 (12)
C19—P1—C1—C2	−45.5 (6)	C10—C11—C12—C7	1.8 (12)
C7—P1—C1—C6	−108.0 (6)	P1—C13—C14—C15	175.4 (7)
C7—P1—C1—C2	78.5 (6)	C18—C13—C14—C15	−1.0 (13)
C13—P1—C1—C2	−164.6 (5)	P1—C13—C18—C17	−175.9 (7)
C1—P1—C7—C12	−162.1 (5)	C14—C13—C18—C17	0.5 (13)
C13—P1—C1—C6	8.8 (6)	C13—C14—C15—C16	0.7 (15)
C19—P1—C1—C6	127.9 (5)	C14—C15—C16—C17	0.1 (16)
C7—P1—C19—C20	84.7 (5)	C15—C16—C17—C18	−0.7 (16)
C13—P1—C19—C20	−39.2 (5)	C16—C17—C18—C13	0.3 (14)
C7—P1—C13—C14	29.7 (8)	Hg1—C19—C20—O1	89.0 (6)
C19—P1—C13—C14	157.0 (6)	Hg1—C19—C20—C21	−91.8 (6)
C1—P1—C13—C18	88.8 (7)	P1—C19—C20—O1	−33.2 (8)
C13—P1—C7—C12	82.2 (6)	P1—C19—C20—C21	146.1 (5)
C19—P1—C7—C12	−43.3 (6)	O1—C20—C21—C22	−8.3 (10)
C1—P1—C19—C20	−154.9 (4)	O1—C20—C21—C26	166.9 (7)
C1—P1—C13—C14	−87.6 (7)	C19—C20—C21—C22	172.4 (7)
C7—P1—C13—C18	−154.0 (7)	C19—C20—C21—C26	−12.4 (10)
C19—P1—C13—C18	−26.7 (8)	C20—C21—C22—C23	176.0 (9)
C1—P1—C7—C8	19.3 (6)	C26—C21—C22—C23	0.5 (13)
C13—P1—C19—Hg1	−159.0 (3)	C20—C21—C26—C25	−175.1 (7)
C13—P1—C7—C8	−96.3 (6)	C22—C21—C26—C25	0.0 (11)
C7—P1—C19—Hg1	−35.2 (4)	C21—C22—C23—C24	−0.2 (16)
C1—P1—C19—Hg1	85.3 (3)	C22—C23—C24—F1	−179.5 (9)
C19—P1—C7—C8	138.1 (5)	C22—C23—C24—C25	−0.8 (17)
C2—C1—C6—C5	−0.6 (11)	F1—C24—C25—C26	−180.0 (8)
P1—C1—C6—C5	−174.0 (6)	C23—C24—C25—C26	1.3 (15)
C6—C1—C2—C3	−0.5 (11)	C24—C25—C26—C21	−0.9 (12)

Symmetry codes: (i) −*x*+1, −*y*+1, −*z*+1; (ii) −*x*+1, −*y*+2, −*z*+1; (iii) −*x*+2, −*y*+2, −*z*+1; (iv) *x*−1, *y*−1, *z*; (v) *x*−1, *y*, *z*; (vi) *x*+1, *y*, *z*; (vii) −*x*+1, −*y*+2, −*z*; (viii) *x*+1, *y*+1, *z*; (ix) −*x*+1, −*y*+1, −*z*; (x) −*x*, −*y*+1, −*z*.

### Hydrogen-bond geometry (Å, °)

**Table:**

*D*—H···*A*	*D*—H	H···*A*	*D*···*A*	*D*—H···*A*
C4—H4···O1^vi^	0.93	2.59	3.271 (11)	131
C12—H12···O1	0.93	2.32	3.124 (8)	144
C22—H22···O1	0.93	2.44	2.749 (10)	100

Symmetry codes: (vi) *x*+1, *y*, *z*.
